# New Candidate Biomarkers in the Female Genital Tract to Evaluate Microbicide Toxicity

**DOI:** 10.1371/journal.pone.0110980

**Published:** 2014-10-21

**Authors:** Scott Fields, Benben Song, Bareza Rasoul, Julie Fong, Melissa G. Works, Kenneth Shew, Ying Yiu, Jon Mirsalis, Annalisa D'Andrea

**Affiliations:** Biosciences Division, SRI International, Menlo Park, California, United States of America; Centers for Disease Control and Prevention, United States of America

## Abstract

Vaginal microbicides hold great promise for the prevention of viral diseases like HIV, but the failure of several microbicide candidates in clinical trials has raised important questions regarding the parameters to be evaluated to determine in vivo efficacy in humans. Clinical trials of the candidate microbicides nonoxynol-9 (N9) and cellulose sulfate revealed an increase in HIV infection, vaginal inflammation, and recruitment of HIV susceptible lymphocytes, highlighting the need to identify biomarkers that can accurately predict microbicide toxicity early in preclinical development and in human trials. We used quantitative proteomics and RT-PCR approaches in mice and rabbits to identify protein changes in vaginal fluid and tissue in response to treatment with N9 or benzalkonium chloride (BZK). We compared changes generated with N9 and BZK treatment to the changes generated in response to tenofovir gel, a candidate microbicide that holds promise as a safe and effective microbicide. Both compounds down regulated mucin 5 subtype B, and peptidoglycan recognition protein 1 in vaginal tissue; however, mucosal brush samples also showed upregulation of plasma proteins fibrinogen, plasminogen, apolipoprotein A-1, and apolipoprotein C-1, which may be a response to the erosive nature of N9 and BZK. Additional proteins down-regulated in vaginal tissue by N9 or BZK treatment include CD166 antigen, olfactomedin-4, and anterior gradient protein 2 homolog. We also observed increases in the expression of C-C chemokines CCL3, CCL5, and CCL7 in response to treatment. There was concordance in expression level changes for several of these proteins using both the mouse and rabbit models. Using a human vaginal epithelial cell line, the expression of mucin 5 subtype B and olfactomedin-4 were down-regulated in response to N9, suggesting these markers could apply to humans. These data identifies new proteins that after further validation could become part of a panel of biomarkers to effectively evaluate microbicide toxicity.

## Introduction

Topical microbicides have been proposed as agents to prevent the transmission of HIV by creating chemical, biological, and/or physical barriers to infection, or by blocking or inactivating the virus at the mucosal surface where infection can occur. An ideal microbicide would need to demonstrate both protection against HIV infection and low toxicity after repeated use. Although several candidate microbicides initially appeared promising in preclinical safety studies, they later proved to be ineffective in clinical trials [1]–[13]. In some cases, they actually increased the risk of infection, e.g. cellulose sulfate [12]. Similarly, nonoxynol-9 (N9), a contraceptive spermicide that has previously been shown to be safe in preclinical and phase I studies, generated disappointing clinical data as a protective microbicide [11], [13]. In fact, repetitive use of N9 resulted in genital irritation/inflammation and increased risk of acquiring HIV [11]. The limited success of putative microbicides in clinical trials demonstrates a need for better parameters to predict the safety of candidates undergoing preclinical development. One approach is to develop a robust series of biomarkers capable of predicting cellular and molecular changes occurring in the vaginal mucosa/epithelium during microbicide treatment. Such markers could have utility both in preclinical development and, eventually, in clinical development as well.

The current preferred pre-clinical model for assessment of microbicide safety is the rabbit vaginal irritation (RVI) model [14]–[20]. The assay requires euthanizing all study animals, endpoints of the RVI are mostly histological, and in the recent years, the limitations of this model in detecting potential toxicity, have clearly demonstrated that additional parameters must be included in the evaluation of new candidate microbicides. Recent studies using this model have identified changes in inflammatory cytokines in vaginal lavage fluids in response to compounds with toxic characteristics [18], [19]. Although the significance of these cytokines has not been fully clarified, they may play a role in creating an environment more susceptible to pathogen infection. For example, the presence of elevated levels of pro-inflammatory factors may increase the proliferation of immune cells in the vaginal tissue parenchyma or increase migration of HIV susceptible immune cells to the vaginal tract.

Although the RVI model has been used for many years to evaluate potential toxic effects of microbicide candidates, it is not without shortcomings. The human and rabbit vaginal tissues show significant structural differences (stratified squamous versus columnar epithelium, respectively) [21], [22] that may be responsible for different susceptibilities to treatment. Moreover, the rabbit lacks cyclic reproductive stages and cervical mucus production, all factors that may affect the environment in the vagina [23].

More recently, mouse vaginal irritation (MVI) models have been developed and used to evaluate microbicide safety [14], [15], [24]–[28]. Advantages of the MVI include a well characterized immune system and a comprehensive protein database that makes it amenable to proteomics studies. In addition, it is significantly less expensive than the RVI model; uses a smaller animal that requires less test articles for safety testing; is not a USDA-regulated species; and provides toxicological results for cross-species comparison, making it an attractive, alternative model for pre-clinical evaluation of vaginal microbicides.

Here, we have used both the RVI and MVI models to identify biomarkers that could be incorporated in the safety evaluations of vaginal anti-HIV microbicides. For our studies we selected to use two known vaginal irritants [18], [29], the spermicide N9 and the antiseptic benzalkonium chloride (BZK) as models of toxic compounds for vaginal administration, and we identified vaginal proteins that were altered following application of these compounds.

## Materials and Methods

### Reagents

Nonoxynol-9 (N9) was purchased from Spectrum Chemicals and Laboratory Products (Gardena, CA); tenofovir was purchased from AK Scientific (Union City, CA); benzalkonium chloride (BZK) and high viscosity sodium carboxymethylcellulose (CMC) were purchased from Sigma Aldrich (Saint Louis, MO); mouse and rabbit apolipoprotein A-I, apolipoprotein C-1, CD166, and rabbit IL-8 ELISA kits were purchased from EIAab (Wuhan, China); mouse fibrinogen ELISA kit was purchased from Abcam (Cambridge, MA); mouse plasminogen ELISA kit was purchased from GenWay (San Diego, CA); mouse IL-8 ELISA kit was purchased from MyBioSource (San Diego, CA); rabbit plasminogen ELISA kit was purchased from Alpco (Salem, NH); rabbit fibrinogen ELISA kit was purchased from Molecular Innovations (Novi, MI); medroxyprogesteron acetate was purchased from Pharmacia (New York, NY); CellTiter-Glo was purchased from Promega (Madison, WI); RNeasy kits were purchased from Qiagen (Valencia, CA); rabbit anti-human olfactomedin 4 (OLFM-4) polyclonal antibody was purchased from Abcam (Cambridge, MA); rabbit anti-human mucin 5B polyclonal antibody was purchased from Bioss USA Antibodies (Woburn, MA); rabbit anti-mouse CD166 was purchased from GeneTex (Irvine, CA); rabbit anti-mouse β-actin was purchased from Abcam (Cambridge, MA); IRDye 800 Goat anti-rabbit IgG was purchased from Li-Cor (Lincoln, NE); mouse anti-human β-actin monoclonal antibody was purchased from Cell Signaling Technology (Danvers, MA); donkey anti-mouse IgG antibody conjugated to IRDye 680 LT and goat anti-rabbit IgG antibody conjugated to IRDye 800CW were purchased from LI-COR biosciences (Lincoln, NE). Precast 4–12% Bis-Tris SDS-PAGE, 3–8% Tris-acetate SDS-PAGE gels, and PDVF transfer membranes were purchased from Life Technologies (Grand Island, NY); oligonucleotides were purchased from Integrated DNA Technologies (Coralville, IA).

### Microbicide preparation

8% N9, and 1% tenofovir were prepared in 2% sodium carboxymethylcellulose, and 2% BZK was prepared in ultrapure water. Vehicle, N9, and BZK microbicides were prepared no more than three days prior to use and were stored at 4°C. Prior to dosing, microbicides were allowed to reach ambient temperature. Buffergel, was obtained from ReProtect Inc. in its clinical formulation, and was stored at room temperature until use.

### Cell lines and challenge with N9

Vk2 (E6/E7) cells (CRL-2616) were obtained from American Type Culture Collection (Rockville, MD). Cells were grown in keratinocyte-serum free medium (K-SFM) containing 5 ng/ml recombinant epidermal growth factor and 50 µg/ml bovine pituitary extract all purchased from Invitrogen Corporation (Grand Island, NY). A maximum tolerated dose of N9 was determined by serially diluting the microbicide from 1% to 0.001% in cell culture medium and each dilution added to individual wells of an 80–90% confluent monolayer of Vk2 Cells in 96-well plates. Cell viability was performed using CellTiter-Glo.

### Quantitative RT-PCR

Frozen vaginas were minced and placed in RNAlater (Life Technologies, Grand Island, NY) and stored at −20°C until processed. Total RNA was isolated (RNeasy kit) from vaginal tissues of 3 animals per treatment group using Ambion *mir*Vana Kit (Life Technologies, Grand Island, NY). cDNA was prepared using Superscript RTII (Life Technologies, Grand Island, NY). Quantitative RT-PCR was conducted using the LightCycler 480 RT-PCR machine and SYBR green I master mix (Roche, Indianapolis, IN). RT-PCR oligos used are shown in **Table S1**. The threshold cycle for each test gene and glyceraldehyde 3-phosphate dehydrogenase (GADPH) was determined and an average of threshold cycles was calculated for each test gene. Each test gene's average threshold cycle was normalized to the GADPH average threshold cycle and expression relative to GADPH was reported as 2^“Ct” GAPDH – Ct gene^ (where “Ct” indicates cycle threshold).

### Immunoblot analysis of mouse and human proteins

Mouse proteins were extracted from vaginal tissues using SDS extraction buffer (50 mM Tris pH 7.5, 0.5% SDS, Halt Protease inhibitor (Pierce, Rockford, IL) for 1.5 hours on ice after vortexing with 0.5 mm glass beads (Next Advance, Averill Park, NY) five times with 1 min vortexing and 1 min rests. Protein concentration was measured using a Nanodrop spectrophotometer (Thermo Scientific, Rockford, IL) and BCA assay (Pierce, Rockford, IL). Protein lysates (50 µg) were resolved on a 10% SDS-Page gel. The gel was transferred (wet transfer at 300 mA) to a nitrocellulose membrane and then blocked with 5% non-fat dry milk in 1× PBS. For the detection of CD166 antigen in mouse samples, the primary antibody was applied in blocking buffer overnight at 4°C: Secondary antibody was used incubated at room temperature for 1 hour. Following extensive washing, the membranes were scanned using the LI-COR Odyssey imaging system.

To detect OLFM-4 and β-actin, Vk2 cell extracts were resolved on 4–12% Bis-Tris SDS-PAGE gels and mucin 5B was resolved on 3–8% Tris-acetate SDS-PAGE gels f. Gels were transferred to PDVF membranes and probed with the appropriate primary and secondary antibodies and detected using the LI-COR Odyssey imaging system.

### Animals

All animal experiments were reviewed and approved by the Institutional Animal Care and Use Committee (IACUC) at SRI International and were performed in accordance with relevant guidelines and regulations in a facility accredited by the Association for the Assessment and Accreditation of Laboratory Animal Care International (AAALAC).

### Mouse vaginal irritation studies

Female CD1 mice were purchased from Harlan Laboratories (Livermore, CA) and were housed in hanging polypropylene cages with hardwood chip bedding; Purina rodent chow #5002 and reverse osmosis purified water were provided *ad libitum*. All mice (7–9 weeks of age) were hormonally synchronized with two subcutaneous doses of 3 mg of medroxyprogesterone acetate on days −8 and −1. The mice were randomized into treatment groups consisting of between 6 and 12 animals per group per study and were dosed daily for 11 days with 50 µl of the microbicide candidates using a luer-lock syringe attached to a 20-gauge applicator inserted 0.8 cm into the vagina. Mouse vaginal brush collection (7 hours after dosing) was done using MicroBrush X (MicroBrush International, Grafton, WI). Brushes were pre-wetted in saline and inserted approximately 0.8 cm into the vaginal cavity. The brushes were turned 90 degrees clockwise and counterclockwise three times, removed, and placed in saline containing 1× protease inhibitor cocktail (Thermo Scientific). Any visual presence of blood was noted but not scored. Vaginal brush samples were collected on days 7 and 10. For the first two studies, a single brush sample per mouse per collection day was obtained, but on the last two studies, three brushes were used per mouse per collection day to maximize the yield of sample recovery. Each individual brush was placed into 0.1 ml of 1× PBS pH 7.6 plus 1× protease inhibitors (Thermo Scientific). The brushes were vortexed in the collection buffer and in the cases where multiple brushes were used, the samples were pooled, mixed, aliquoted, and snap frozen in dry ice/ethanol prior to storage at −80°C. Vaginas were harvested on the final day of the study and snap frozen using dry ice/ethanol baths prior to storage at −80°C.

### Rabbit vaginal irritation studies

Female white New Zealand White rabbits (20–28 weeks of age) were obtained from Harlan Laboratories; and were housed individually in stainless steel cages; Teklad Rabbit Diet and reverse osmosis purified water were provided *ad libitum*. Animals were randomized into treatment groups consisting of 6 animals per group per study and one day prior to dosing the vaginal cavities were washed with 1 ml sterile 0.9% saline solution. The rabbits were dosed daily for 10 days with 1 ml of the microbicide candidates using a syringe attached to a lightly curved 6 inch stainless steel ball-tipped cannula that was inserted approximately 5–6 inches into the vagina. On days 7 and 10 (collection 7 hours after last dose), sterile cotton tipped swabs were used to collect vaginal cavity material. Three cotton swabs were used per rabbit per collection day. Each individual swab was placed into 0.5 ml of 1× PBS pH 7.6 plus 1× protease inhibitors (Thermo Scientific). The swabs were vortexed in the collection buffer and all three collection tubes per rabbit were pooled, mixed, aliquoted, and snap frozen in liquid nitrogen prior to storage at −80°C.

### ELISA assays

Vaginal brush samples were thawed and tested using the individual kit's instructions. Briefly, each dilution of the standard was run in triplicate and samples were run in duplicate. The OD settings for each ELISA were set by the manufacturer. Values above or below the standard curve are reported as having a value equal to the highest or lowest value in the standard for the kit. Plates were scanned using a Spectramax 2 plate reader (Molecular Devices, Sunnyvale, CA). Generation of standard curves, determination of unknown protein concentrations, and statistical calculations were accomplished using GraphPad Prism software.

### Vaginal tissue protein extraction for proteomic analysis

Vaginal tissue samples were thawed on ice, weighted, and washed with PBS buffer. For tissue protein extraction, approximately 100 mg vaginal tissue was minced into 2 mm pieces in 1 ml lysis buffer (50 mM Tris, pH 7.4, 0.5% SDS, EDTA-free protease inhibitor cocktail). Approximately 0.2 ml glass beads (0.5 mm, Sigma-Aldrich, St. Louis, MO) were added to each sample. Subsequently, samples were homogenized 5 times with 1 min for each time by a Bullet-Blender (Next Advance, Averill Park, NY) at 4°C and then left on ice for 1 hour. Debris was removed by centrifugation at 12,000 g for 15 min at 4°C, and supernatants were stored at −80°C for proteomics sample preparation. Total protein of the samples was measured using a NanoDrop 2000 (Thermo Scientific, USA).

### Proteomics sample preparation and TMT labeling

Filter-aided sample preparation (FASP) using 10k cutoff filters, developed by Mann [30], was applied to purify and digest both brush samples and tissue protein lysates. Briefly, 600 µL brush sample or 200 µL tissue protein lysate was transferred into a 1.5 mL Microcon YM-10 centrifugal unit (Millipore, Billerica, MA, USA). Protein reduction, alkylation, and tryptic digestion were performed step by step in the centrifugal unit. After overnight tryptic digestion at 37°C, the peptides were eluted twice with 150 µL 50 mM ammonium bicarbonate. The total protein or peptide concentration in each step was measured using NanoDrop 2000. The eluted peptides were dried by vacuum centrifugation and then resuspended in 50 mM Tetraethylammonium bromide (TEAB) for Tandem Mass Tag (TMT) labeling.

50 µg digested peptides from each tissue sample and 10 µg peptides from each brush sample were incubated with an amine-reactive 6-plex TMT tag (Thermo Scientific, USA) for 2 hours at room temperature. In mouse studies 1 and 2, the digested peptides from brush samples from the same group of animals were pooled together for TMT labeling due to low peptide content. Reactions were quenched by adding 8 µL 5% hydroxylamine and incubated at room temperature for 15 min. The labeled peptides were then combined together and excess TMT tags were removed by a 3×8 mm SCX trap column then desalted by a 3×8 mm C18 RP trap column (Bruker-Michrom, CA, USA). Purified labeled peptides were dried by vacuum centrifugation and then resuspended in 0.1% formic acid (FA) for Multidimensional Protein Identification Technology (MudPIT) analysis.

### Nano-LC-MS/MS

Each TMT labeled sample was separated using a nano-LC system (Agilent 1200, Palo Alto, CA, USA) and analyzed with an LTQ-XL Orbitrap ETD (Thermo Fisher Scientific, San Jose, CA, USA) equipped with a nano-ESI source. Full MS spectra were acquired in positive mode over a 350–1800 *m/z* range, followed by four CID (collision induced dissociation) and HCD (higher-energy collisional dissociation) events on the four most intense ions selected from the full MS spectrum and using a dynamic exclusion time of 30 s [31]. Four CID scans (maximum inject time 100 ms, minimum signal threshold 500 counts, collision energy 35%, activation time 30 ms, isolation width 1.0 *m/z*) were used for peptide identification and four corresponding HCD scans (maximum inject time 300 ms, minimum signal threshold 500 counts, collision energy 45%, activation time 30 ms, isolation width 1.0 *m/z*) were used for quantitation.

### Database Search

Acquired tandem mass spectra were searched against the European Bioinformatics Institute International Protein Index mouse protein database (version 3.73). A decoy database containing the reverse sequence of all the proteins was appended to estimate false discovery rate (FDR). The search was performed using SEQUEST algorithm incorporated in Proteome Discoverer 1.3.0.339 (Thermo Finnigan, CA, USA). The precursor mass accuracy was limited to 13 ppm and fragment ion mass tolerance was set at 1.1 Da. Fully tryptic enzyme specificity and up to two missed cleavages were allowed. Fixed modifications included carbamidomethylation on cysteines and variable modifications included oxidation on methionines and TMT adduction to peptide N-termini, lysines, and tyrosines. Peptide quantitation was also performed in Proteome Discoverer 1.3.0.339 in the same workflow. A TMT 6-plex quantitation method was used for HCD-based quantitation. Mass tolerance was set at 150 ppm for reporter TMT tags. The intensity of each peptide was normalized to protein median intensity before calculating the ratio of different tags from the same peptides. Protein quantitation was calculated based on the data of each quantified peptide.

### Statistical analysis

For ELISAs and RT-PCR assays, Student's T-tests were conducted with Welch's correction for compound groups versus vehicle controls. P values ≤0.05 are considered.

For proteomics analysis, FDR calculated through a decoy database search was set as 0.05 for both protein identification and quantitation. For each study, standard deviation was calculated for the protein level alteration in every group.

## Results

### Proteomic analysis of mouse vaginal brush and tissue samples reveal changes in several proteins after treatment with N9 and BZK

To identify protein changes induced by vaginal microbicide candidates with an undesirable safety profile, mice were treated with either vehicle, 8% N9, 2% BZK, or 1% tenofovir (a non-toxic microbicide) [32] for 10 consecutive days (as per standard preclinical evaluations in mice and rabbits [23]), with mucosal samples and tissue samples collected at day 7 and 10 for analysis by 2D LC-MS/MS, respectively.

#### Proteins from vaginal brushes

**Figure 1:**
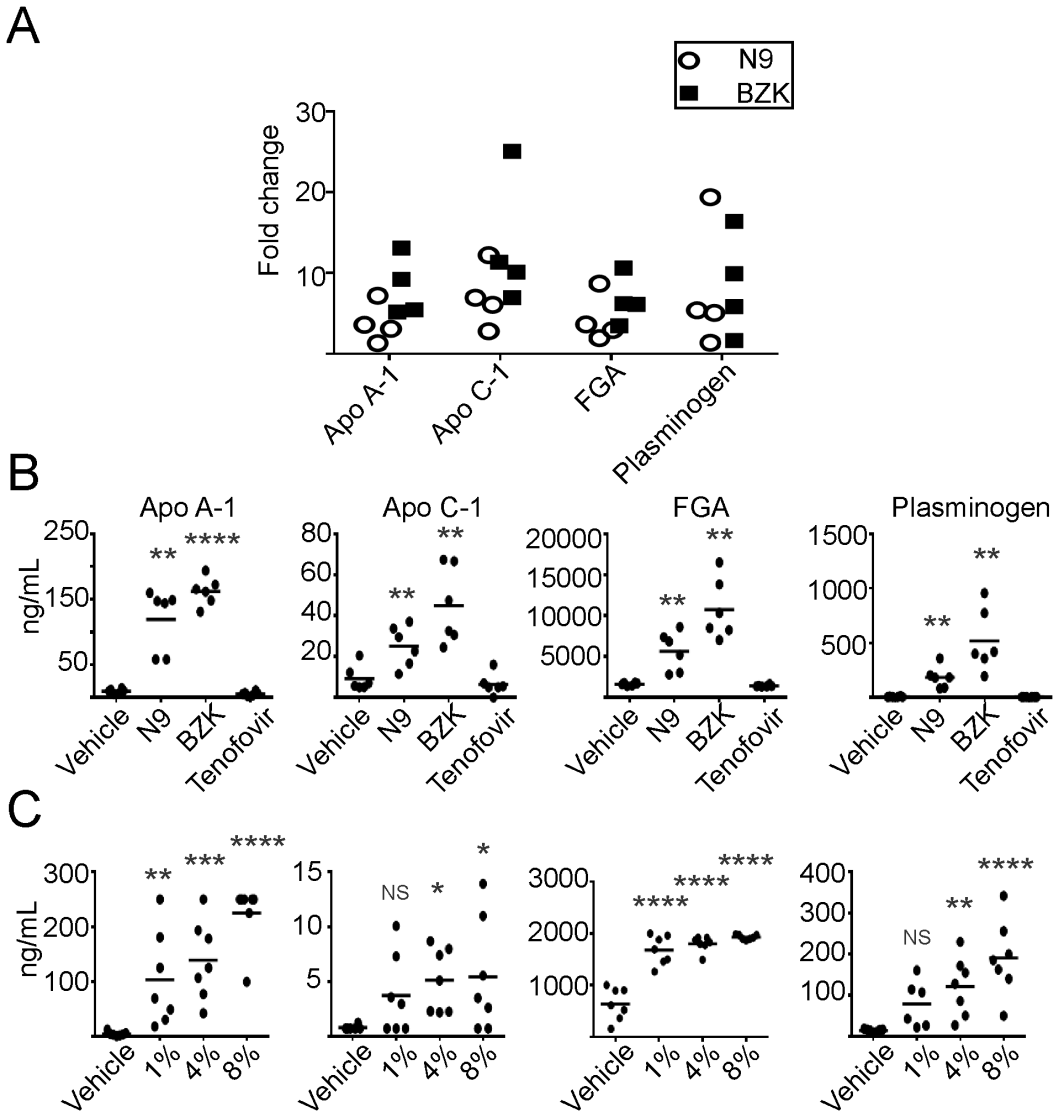
Quantitative proteomic analysis of mouse brush samples. (A) LC-MS/MS analysis of mouse proteins in vaginal brush samples that are affected by treatment with N9 (open, black circles) or BZK (closed squares). Shown are proteins that are increased compared to vehicle controls. The following proteins are depicted in the graph: fibrinogen alpha polypeptide isoform 2 (FGA), plasminogen (PLG), apolipoprotein A-1 (Apo A-1), and apolipoprotein C-1 (Apo C-1). Each data point represents the average of three replicates per individual experimental study. Only proteins shown to change in four studies are represented. (B) ELISA analysis of mouse proteins in vaginal brush samples that are increased in response to treatment with 8% N9 or 2% BZK. Statistical analysis was performed using a two-tailed Student's T-test of N9 and BZK samples compared to vehicle controls (**** = p≤0.0001, ** = p≤0.01). (C) ELISA analysis of mouse proteins in vaginal brush samples that are increased in response to treatment with vehicle, 1% N9, 4% N9, or 8% N9. Statistical analysis was performed using a two-tailed student's t-test of N9 and BZK samples compared to vehicle controls (**** = p≤0.0001, *** = p≤0.001, ** = p≤0.01, * = p≤0.05, NS = not significant).

**Figure 2:**
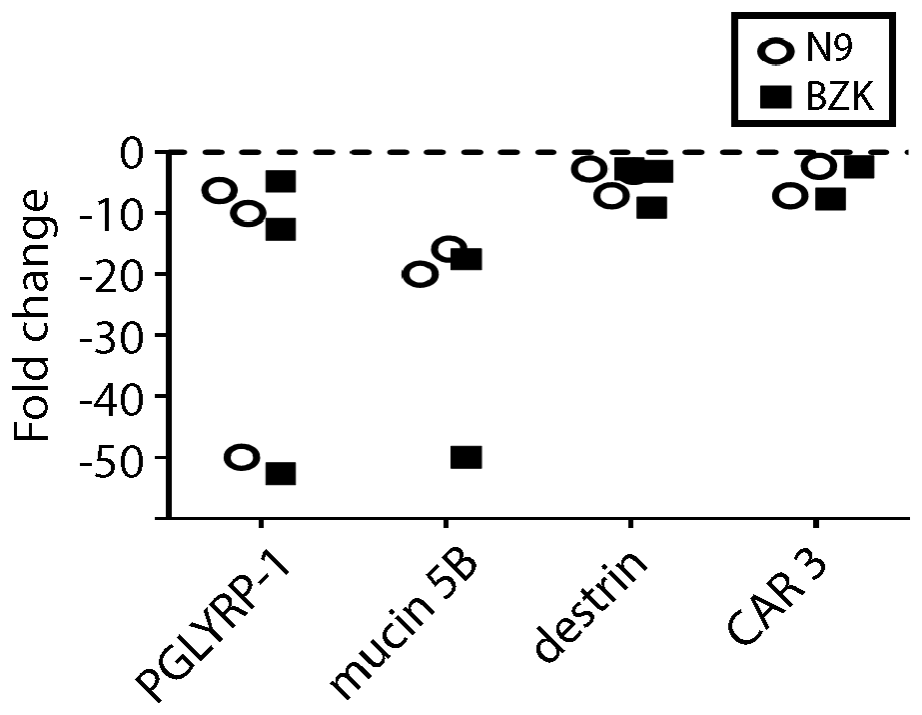
LC MS/MS proteomic analysis of mouse proteins in vaginal brush samples that are down-regulated in response to treatment with N9 (open, black circles) or BZK (closed squares). Shown are proteins that were decreased relative to vehicle controls. The following proteins are shown: peptidoglycan recognition protein 1 (PGLYRP-1), mucin 5 subtype B (mucin 5B), destrin, and carbonyl reductase 3 (CAR3). Each data point represents the average of three replicates per individual experimental study. Only proteins shown to change in a minimum of two studies are represented.

Changes in proteins present in mucosal secretions upon treatment with toxic compounds can be exploited to define biomarkers of toxicity. In this study, mucosal secretions were collected by brush sampling. Proteins that showed a consistent >2-fold change relative to vehicle control in at least two studies were selected for further analysis. A total of 1017 proteins were identified with a false discovery rate (FDR) cutoff of 0.05. After 7 days exposure to toxic microbicides, a total of 10 proteins were selected for further analysis (**Table 1**). Six proteins were increased upon exposure to N9 and BZK, while 4 proteins decreased. Notably the increased proteins are thought to be serum derived, probably as a result of exudates due to the erosive nature of the microbicides. These consisted of apolipoprotein A-1 (Apo A-1; range change 2 to 7-fold for N9 and 5 to 13-fold for BZK), apolipoprotein C-1 (Apo C-1; range change 3 to 12-fold and 3 to 12-fold for BZK), fibrinogen alpha polypeptide isoform 2 (FGA; range change 3 to 9-fold and 2 to 8-fold for BZK), plasminogen (PLG; range change 2 to 19-fold and 2 to 8-fold for BZK), heat shock cognate 71 kDa protein (HSPA8; range change 3 to 4-fold for both N9 and BZK), and Corticosteroid-binding globulin (SERPINA6; range change 2 to 21-fold and 3 to 31-fold for BZK), respectively (**Figure 1A** and **Table 1**). In contrast the following four proteins were decreased in vaginal brush samples (**Table 1** and **Figure 2**): peptidoglycan recognition protein 1 (PGLYRP-1; range change 6 to 50-fold for N9 and 5 to 52–fold for BZK), mucin 5 subtype B (mucin 5B, range change 16 to 20-fold for N9 and 20 to 50-fold for BZK), destrin (DSTN, range change 2.5 to 7-fold for N9 and 3 to 10-fold for BZK), and carbonyl reductase 3 (CAR3, range change 2.5 to 7-fold for N9 and 2 to 10-fold for BZK). These proteins are produced by vaginal tissue and are thought to confer protection against microorganisms.

**Table 1:** Summary of proteins whose levels significantly altered upon N9 or BZK treatment determined by proteomic analysis in mouse vaginal brush and tissue samples.

Vaginal brush samples (day7)	Gene name	N†	N9		BZK		Tenofovir
			avg*	std	avg	std	
Apolipoprotein A-1	APOA1	4	3.80	2.46	8.22	3.73	0.7
Apolipoprotein C-1	APOC1	4	7.01	3.91	13.36	8.02	0.22
Fibrinogen, alpha polypeptide isoform 2	FGA	4	4.30	3.00	6.60	2.96	1.23
Plasminogen	PLG	4	7.81	7.94	8.44	6.28	0.77
Heat shock cognate 71 kDa protein	HSPA8	2	3.62	0.02	3.87	0.22	0.83
Corticosteroid-binding globulin	SERPINA6	3	11.17	10.06	17.08	14.67	0.35
**Peptidoglycan recognition protein 1** ††	PGLYRP-1	3	0.09	0.07	0.10	0.10	3
**Mucin 5, subtype B, tracheobronchial**	Muc5B	2	0.06	0.01	0.04	0.03	1.07
Destrin	DSTN	3	0.28	0.12	0.27	0.14	1.64
Carbonyl reductase 3	CAR3	2	0.29	0.21	0.28	0.21	1.48

Vaginal tissue samples	Gene name	N†	N9		BZK		Tenofovir
			avg	std	avg	std	
**Peptidoglycan recognition protein 1**	PGLYRP-1	2	0.39	0.07	0.43	0.16	ND
CD166 antigen	CD166	2	0.47	0.07	0.4	0.01	ND
**Mucin 5, subtype B, tracheobronchial**	Mucin 5B	3	0.43	0.05	0.30	0.15	0.66
Olfactomedin-4	OLFM-4	3	0.49	0.35	0.39	0.31	0.99
Anterior gradient protein 2 homolog	AGR2	3	0.41	0.15	0.31	0.26	0.94
Calcium-activated chloride channel regulator 1	CLCA2	2	0.39	0.16	0.26	0.02	0.62
Protein-glutamine gamma-glutamyltransferase 2	TGM2	3	0.55	0.35	0.58	0.28	0.66

*Average signal compared to vehicle controls which are set to a default value of 1,

† number of studies where protein was identified (for both N9 and BZK), ND = not detected,

†† proteins shown in bold are identified in both brush and tissue samples.

Tenofovir treatment had no significant effect on the expression levels of those proteins, with the exception of Apo C-1 and PGLYRP-1, which were decreased 4-fold and increased 3-fold in vaginal brush samples, respectively. A summary of their known or putative biological functions of the proteins affected by N9 and BZK treatment is listed in **Table S2**.

Proteins that were consistently upregulated in four replicate proteomic experiments (**Figure 1A**) were then confirmed by ELISA analysis. Upon exposure to N9, Apo A-1 was increased an average of 12-fold over vehicle (ranging from a 4 to 29-fold increase) or tenofovir control and plasminogen was elevated an average of 27-fold (ranging from an 18 to 71-fold increase). Both Apo C-1 and fibrinogen alpha polypeptide isoform 2 (FGA) showed a lesser increase, averaging ∼3-fold each (ranging from a 0.53 decrease to a 7.4-fold increase and a 1.55 to 6 fold-increase, respectively) (**Figure 1B**). Levels of these proteins were even higher following treatment with BZK when compared with vehicle/tenofovir control treatment. Apo A-1 showed a 17-fold increase (range 9–36-fold); plasminogen increased 76-fold (range 15 to 191-fold), Apo C-1 increased 5-fold (range 1 to 13-fold), and FGA showed a 7-fold increases, (range 4 to 12-fold), respectively. The biological function of these proteins is summarized in **Table S2**. The identified proteins are all serum proteins possibly present in the brush sample due to the tissue damaging effect of N9 and BZK, which in few circumstances caused bleeding in the vaginal cavity (not shown). Blood was predominantly found in brush samples in mice and rabbits treated with BZK, but was only rarely observed in animals (mice or rabbits) treated with N9, emphasizing that the presence of blood could not be used as a predictor to detect toxic effects of test articles. To cover a range of concentrations of N9 that were representative of the dose used in clinical trials (3.5%) and to reduce the disruption of the cervical epithelium, we examined the levels of Apo A-1, Apo C-1, FGA, and plasminogen using lower concentrations of N9 (1%, 4%, and 8%). With the exception of Apo C-1, N9 exhibited a dose dependent increase of each marker in vaginal brush samples. Apo A-1 and FGA showed a marked increase with as little 1% N9 and all 4 proteins were elevated with 4% N9 which increased further with 8% N9 (**Figure 1C**). These data suggest that these proteins could be incorporated into a rapid and sensitive set of biomarkers for the evaluation of microbicide toxicity. In addition, one experiment was conducted with a clinical trial formulation of Buffergel, a microbicide that was safe but failed to protect women against HIV. Buffergel treatment, like tenofovir did not demonstrate an increase in these proteins when tested by ELISA (**Figure S1**).

#### Proteins from Vaginal Tissues

**Figure 3:**
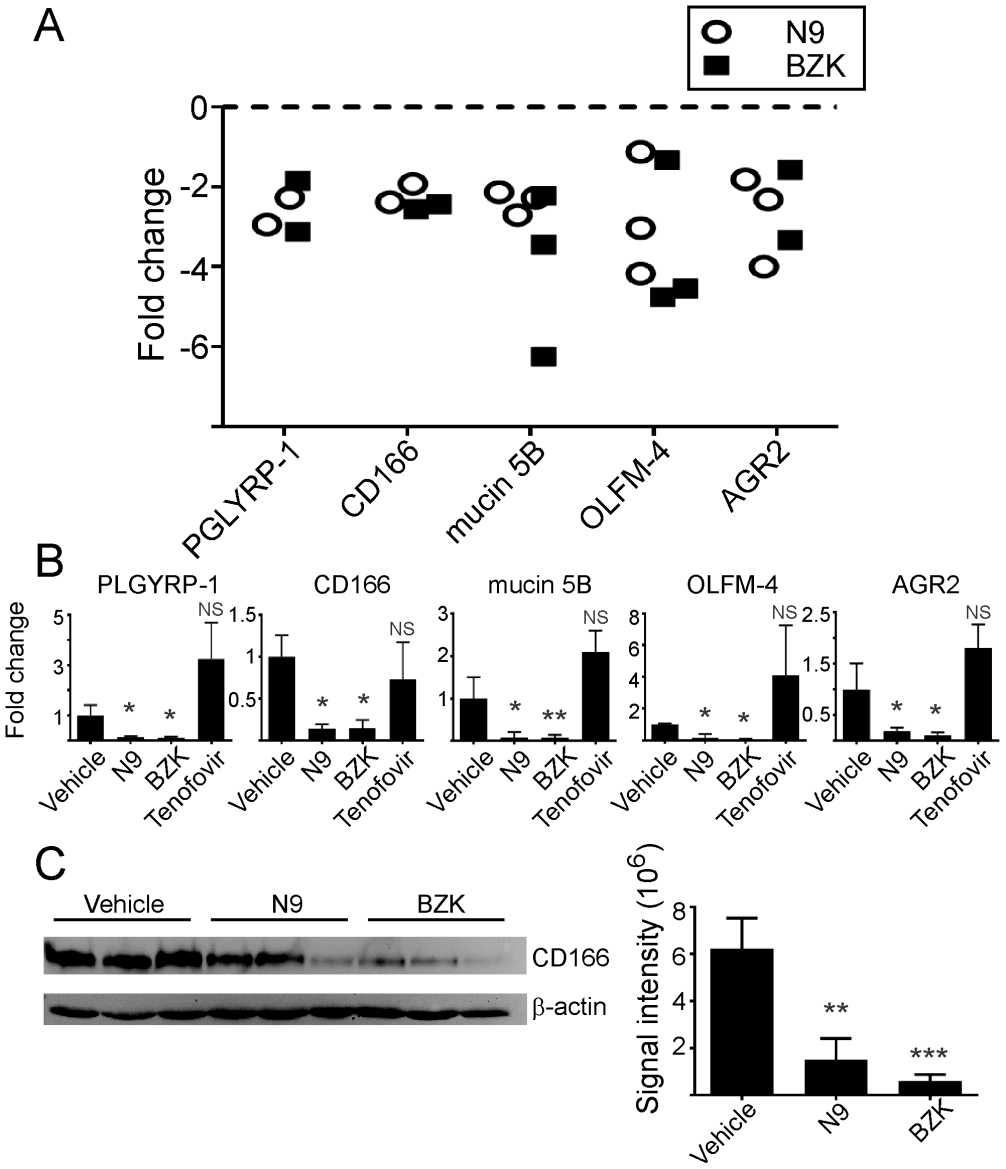
Quantitative proteomic and RT-PCR analysis of mouse tissue samples. (A) LC MS/MS proteomic analysis of mouse vaginal tissue samples down-regulated in response to treatment with N9 (open, black circles) or BZK (black squares). The following proteins are depicted in the graphs: peptidoglycan recognition protein 1 (PGLYRP-1), CD166 antigen (CD166), mucin 5 subtype B (mucin 5B), olfactomedin-4 (OLFM-4), and anterior gradient protein 2 homolog (AGR2). Each data point represents the average of three replicates per individual experimental study. Only proteins shown to change in a minimum of two studies are represented. (B) RT-PCR analysis of mouse mRNA expression. Relative expression levels are normalized to Glyceraldehyde 3-phosphate dehydrogenase (GADPH). Statistical analysis was performed using a two-tailed Student's T-test of N9, BZK, and tenofovir samples compared to vehicle controls (*** = p≤0.001, * = p≤0.05, NS = not significant). (C) Immunoblot of mouse CD166 antigen. Three mice per condition were analyzed. The CD166 signal was normalized to the β-actin signal for quantitation. Statistical analysis was performed using a two-tailed student's T-test of N9 and BZK samples compared to vehicle controls (*** = p≤0.001, ** = p≤0.01).

The use of proteomics for identification of proteins in vaginal fluids revealed several proteins altered by treatment with N9 and BZK; however, focusing only on vaginal secretions limits the potential pool of markers that could be utilized for evaluating microbicide toxicity. Therefore, we proceeded to analyze the changes induced by N9 and BZK in vaginal tissues. Proteomic analysis of vaginal tissue lysates revealed an average of 1,585 unique proteins. Of these, five proteins were found to be consistently down-regulated (average of 2- to 3-fold) (**Table 1** and **Figure 3A**), including PGLYRP-1 (range 2 to 3-fold for both N9 and BZK), CD166 (range 2 to 2.5-fold for N9 and 2.5 to 3-fold for BZK), mucin 5B (range 2 to 2.5-fold for N9 and 2 to 6-fold for BZK), olfactomedin-4 (OLFM-4) (range 1 to 4-fold for N9 and 1 to 5-fold for BZK), and anterior gradient protein 2 homolog (AGR2) (range 2 to 4-fold for N9 and 1 to 15-fold for BZK.

The down-regulation was confirmed by RT-PCR analysis (**Figure 3B**) and CD166 was further confirmed by immunoblot (**Figure 3C**). Changes in mRNA expression levels could be detected for these proteins following treatment with as little as 1% N9 gel (**Figure S1B**). Tenofovir treatment had minimal effect (less than two-fold) on these protein levels in vaginal tissues, suggesting that the changes observed were restricted to compounds with toxic activities. Among all proteins identified from vaginal brushes and tissues, PGLYRP-1 and mucin 5B were the only two proteins affected by treatment in both set of samples. In summary, these results highlight the limited number of proteins in the vaginal tissues that change upon exposure to N9 and BZK, potentially identifying new candidate biomarkers to evaluate safety of candidate microbicides.

### Treatment with N9 and BZK up-regulates the expression of several chemokine genes

**Figure 4:**
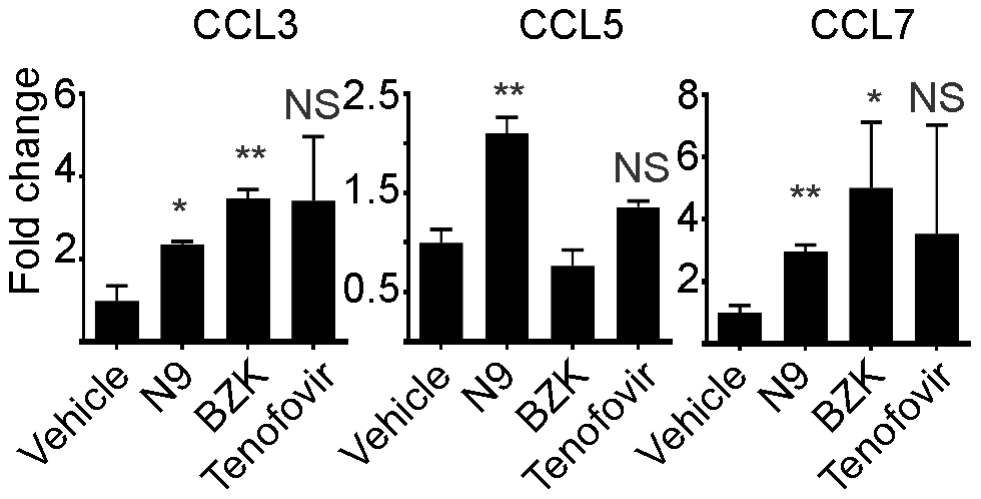
RT-PCR analysis of mouse CCL3, CCL5, and CCL7 gene expression. Relative expression levels are normalized to Glyceraldehyde 3-phosphate dehydrogenase (GADPH). Statistical analysis was performed using a two-tailed Student's T-test of N9, BZK, and Tenofovir samples (3 replicates) compared to vehicle controls (*** = p≤0.001, ** = p≤0.01,* = p≤0.05, NS = not significant).

One of the limitations of our proteomic approach is the difficulty in reliably detecting proteins of low molecular weight (e.g. chemokines). Previously we demonstrated that the chemokine CCL2 (MCP-1) was up-regulated in rabbit brush and tissue samples when treated with N9 and BZK [18], and other groups have shown CCL2 and CCL5 (RANTES) to be up-regulated in the MVI model in response to treatment with N9 [33]. To determine if the vaginal levels of additional chemokines were affected by treatment with N9 or BZK, we selected chemokines that had been shown to be upregulated during a preliminary microarray analysis (not shown) and performed RT-PCR for CCL3, CCL5 and CCL7 on rabbit vaginal tissue samples. Our analysis showed upregulated expression of CCL3 and CCL7 in response to N9 or BZK, but not in response to treatment with tenofovir or vehicle (**Figure 4**). While CCL5 was increased upon exposure to N9, as previously reported [33], its expression was not affected by BZK, indicating that CCL5 is not consistently upregulated by all compounds with toxic potential.

### Treatment with N9 and BZK induces similar changes in rabbit brush samples

**Figure 5:**
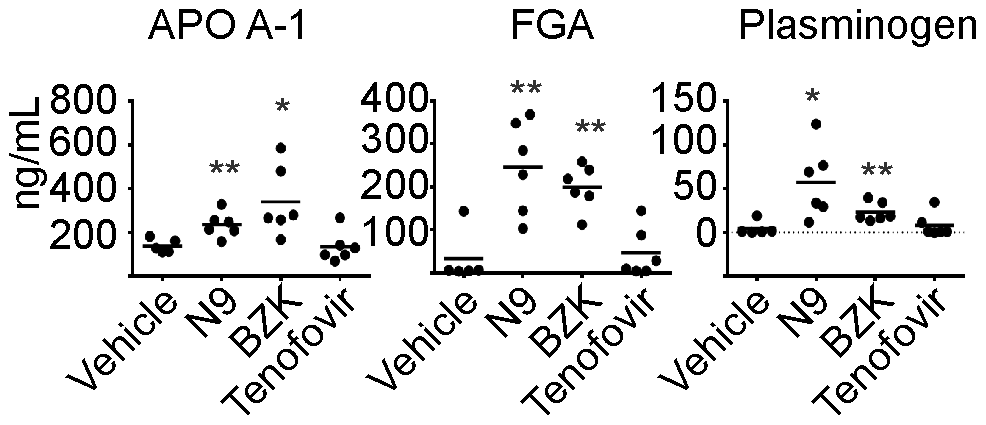
ELISA analysis of rabbit proteins in vaginal brush samples increased in response to treatment with N9 or BZK. Statistical analysis were performed using a two-tailed Student's T-test of N9 and BZK samples compared to vehicle controls (** = p≤0.01, * = p≤0.05, NS = not significant).

Although the RVI model is considered the preferred model for preclinical evaluation of vaginal microbicides, its many limitations have prompted investigators to develop other animal models. The MVI model has been used extensively to examine the efficacy of potential topical microbicides in the prevention of HSV-2 infection [34] and several studies have provided evidence for the MVI model to be a valuable tool for preclinical assessment of toxicity associated with exposure to candidate microbicides [33], [35]. To provide further evidence in support of using the MVI model, we performed a proteomic analysis to determine whether proteins affected by N9 and BZK treatment in mice were consistent across species. For this evaluation, rabbits were treated with the same concentrations of N9 and BZK used in the mouse studies, and brush samples were analyzed. Our proteomic analysis from N9 or BZK treated samples showed a concordance between mouse and rabbit with upregulation of FGA, Apo A-1, Apo C-1, plasminogen, and corticosteroid binding protein (**Table 2**). ELISA assays confirmed the changes observed by proteomics analysis for FGA, Apo A-1, and plasminogen in brush samples (**Figure 5**), with no significant effect observed upon treatment with tenofovir. Apo C-1 showed a similar trend although the changes were not statistically significant (not shown). The lack of reagents specific for rabbit proteins impeded the testing of additional markers. In addition, the proteomics analysis also confirmed the down-regulatory effect of N9 and BZK on destrin and proteins from the mucin family; in rabbit we detected down-regulation of mucin 1 rather than mucin 5B. Some of the other proteins affected by N9 and BZK in MVI model (such as CAR3, and heat shock cognate 71 kDa protein) were not identified in our proteomic analysis in the rabbit study, and we see two possible explanations. In the first case, the anatomy of rabbits may play a critical role as the bladder empties into the vagina, therefore diluting all proteins or test articles present in the vagina. As a consequence, the residence time of the test articles can be significantly impacted, therefore masking its real impact on tissues. Alternatively, some of the proteins identified in mice did not appear represented in the rabbit proteomic database and therefore would not be detected using our proteomic approaches.

**Table 2:** Alteration of protein levels in rabbit vaginal brush samples (day 7) after microbicide treatment.

Vaginal brush samples (day7)	Gene name	Treatment Group*								
		N9			BZK			Tenofovir		
		avg*	std	N†	avg	std	N	avg	std	N
**Apolipoprotein A-1** ††	APOA1	17.1	12.5	3	44.2	16.3	3	0.90	0.92	3
**Apolipoprotein C-1**	APOC1	7.4	3.5	3	10.4	4.2	3	0.84	0.14	3
**Fibrinogen, alpha polypeptide isoform 2**	FGA	7.9	0.38	3	29.8	25.5	3	0.61	0.70	3
**Plasminogen**	PLG	5.2	1.2	3	16.5	4.1	3	0.76	0.38	3
**Corticosteroid-binding globulin**	SERPINA6	7.7	3.60	2	4.0	ND	1	0.72	0.11	2
**Peptidoglycan recognition protein 1**	PGLYRP1	2.0	1.07	3	2.9	1.3	2	0.27	0.09	2
Mucin 1	MUC1	0.86	0.61	3	0.29	0.2	3	1.30	0.93	3
**Destrin**	DSTN	0.54	0.28	3	1.0	0.2	3	1.21	0.26	3

* Average signal compared to vehicle controls which are set to a default value of 1,

† number of rabbits where signal was detected in the study,

†† proteins shown in bold are identified in both MVI and RVI models.

### N9 treatment of a human vaginal epithelial cell line up-regulates the mRNA expression levels of proinflammatory proteins while reducing the expression levels of mucin 5B, and OLFM-4

**Figure 6:**
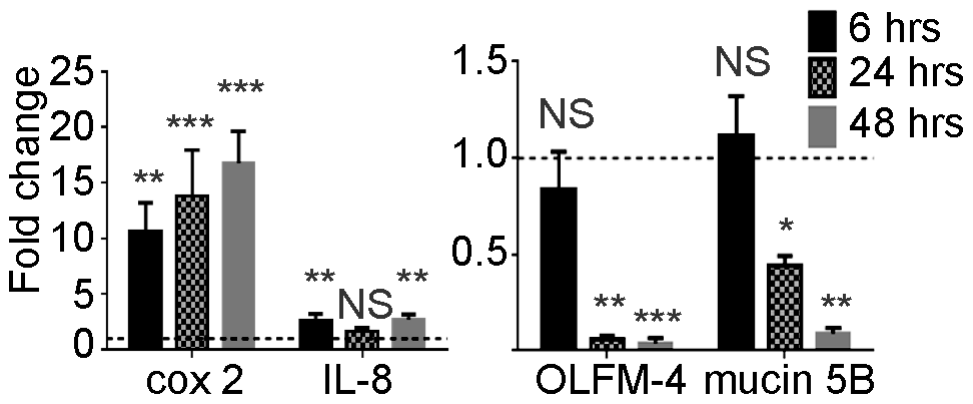
mRNA expression of cyclooxygenase-2 (cox2), Interleukin 8 (IL-8), mucin 5B, and olfactomedin-4 (OLFM-4) in Vκ2 cells treated with N9 for 6, 24, or 48 hours. Shown is the average of two replicate experiments, each sample performed in duplicate for each time point. Also shown is the relative expression levels of N9 treated cells compared to untreated cells, which was set to an expression level of 1 (dashed line). Statistical analysis was performed using a two-tailed Student's T-test of N9 samples compared to untreated controls at each time point (*** = p≤0.001, ** = p≤0.01, * = p≤0.05, NS = not significant).

Due to the difficulty in obtaining human vaginal tissue from patients treated with N9 or BZK, we tested the effects of N9 on cultured human vaginal epithelial cells. For these studies we selected human Vk2 (E6/E7) cells, a vaginal epithelial cell line, frequently used for vaginal studies [36]–[38]. Expression of OLFM-4, mucin 5B and PGLYRP-1 in untreated cells was evaluated using RT-PCR and immunoblots. VK2 cells had detectable levels of mRNA and protein for OLFM-4 and mucin 5B mRNA (**Figure S2**) but not PGLYRP-1 (data not shown). To determine whether N9 may down-regulate the expression of these genes in Vk2 cells, cultures were treated with N9 and transcript changes were monitored following treatment. As was previously demonstrated [38], N9 is highly toxic to Vk2 cells. We used 0.001% N9 as 80% of the cells remained viable after 24 hours at this concentration (**Figure S3**). Expression of OLFM-4 was highly reduced by 24 hours (14.6-fold, ranging from 12.5 to 25-fold), while mucin 5B, although already significantly reduced at 24 hours (2.5 fold, ranging from 2 to 3-fold) showed a total decrease of 12.4-fold (ranging from 9 to 14-fold) at 48 hours (**Figure 6** **, right panel**). As previously reported [38], N9 increased the expression of the pro-inflammatory marker cox2 10-fold (ranging from 8 to 15-fold) within 6 hours of treatment (**Figure 6** **, left panel**) compared to vehicle control cells. We also found that IL-8 was increased approximately 3-fold (ranging from 2 to 4-fold). The data obtained from cell lines suggests that selected proteins identified in proteomic assays in mice and rabbits may in fact serve as predictive biomarker for human studies.

## Discussion

Monitoring changes in the vagina that can efficiently and reliably predict the safety of candidate microbicides is an important step during preclinical and clinical studies of putative new microbicides. For preclinical evaluations, use of the RVI model has long been the primary model to assess the toxicity of vaginal products. This model focuses on histopathological observations and has been repeatedly shown to be less than optimal for preclinical assessments as it is not a good structural model for the human vagina due to the differences in the epithelium (squamous in human versus columnar in rabbits) and position of the urethra inside the vagina. In addition, the study required euthanizing ∼30–35 rabbits for each microbicide evaluated. The unfortunate findings during human clinical trials with N9, and cellulose sulfate (CS), both of which enhanced HIV acquisition [12], [13], have clearly pointed out the need for more comprehensive and meaningful testing.

Although considerable effort has been directed toward the discovery of biomarkers that can be linked to microbicide-induced cervicovaginal inflammation [5], [26], [39], [40], currently there is no FDA approved panel of biomarkers that can be used to demonstrate that changes in specific proteins will increase susceptibility to HIV infection. Identifying proinflammatory markers is a rational approach, and we and others [4], [18], [33] have reported the presence of inflammatory mediators and chemokines that could be responsible for the recruitment of monocytes and T cells in the vaginal milieu. Although the presence of chemokine attractants such as CCL2, CCL3, CCL5, MIP-2, and IL-1β, provides a potential rationale for the increased HIV infection rate observed during clinical trials [13], our data suggests that focusing only on proinflammatory proteins provides an incomplete picture. Considering the complexity of the microenvironment of vagina and the presence of different cytokines under various physiological conditions [40], an attempt to predict microbicide toxicity using cytokines and chemokines alone may not be sufficient. Inclusion of additional biomarkers that can detect changes in the epithelium barrier and local mucosal immunity may provide a more accurate indicator of potential safety issues with a product.

In this study we have used an untargeted LC-MS/MS based quantitative proteomic approach to identify and compare the qualitative and quantitative changes induced by N9 and BZK in both mice and rabbits. Our studies identified a panel of proteins that are altered during treatment with N9 and BZK in the mucosal vaginal environment. Using the MVI model, we identified six proteins (Apo A-1, Apo C-1, FGA, plasminogen, destrin, CAR3), whose levels were increased only in the vaginal brush samples (**Figure 1** **and** **2**). The elevated levels of these proteins are consistent with traces of blood in collected brush samples from some animals (mice and rabbits) treated with N9 and BZK. In addition, three proteins (CD166, OLFM-4, AGR2) were decreased only in the vaginal tissues and two proteins (mucin 5B and PGLYRP-1) were down-regulated in both brush and vaginal tissue samples. These are found on the surface of vaginal epithelia and probably shed into the mucosa. Using ELISA assays we also identified two additional chemokines, CCL3 and CCL7, and confirmed the presence of two previously known chemokines, CCL5 [33] and our previously reported CCL2 [18], as additional proteins that could be added to a potential biomarker panel. By proteomics analysis we didn't consistently detect changes in cytokines or chemokines known to be altered following treatment with N9, fact that could be due to the loss of small size of the proteins during sample preparation procedure. Therefore, we performed RT-PCR to evaluate changes on those small proteins.

Among the down-regulated proteins, OLFM-4, PGLYRP-1 and mucin 5B, have been reported to play important roles in protection against microbial infection [41]–[46]. OLFM-4 is known to be a suppressor of proinflammatory responses in gut epithelium [45], and it has been implicated in maintenance of persistent *Helicobacter pylori* infection where the bacteria use OLFM-4 to suppress the inflammatory response by the intestinal epithelium [45]. Loss of OLFM-4 results in rapid clearance of *H. pylori* due to an aggressive host inflammatory response that includes the release of CCL3, CCL5, and CCL7 [45], chemokines that were identified in this screen. The localized production of these chemokines may be responsible for the recruitment of HIV susceptible monocytes and T cells to sites of vaginal damage induced by the toxic microbicide compounds [18], [33]. Additionally, epithelial cells have been shown to produce chemokines as well as express chemokine receptors [47], [48], which play a role in the migration of immune modulating cells. OLFM-4 may play a similar role in the vagina by suppressing an inflammatory response to vaginal microflora and maintaining proper microbe and tissue homeostasis in the vaginal cavity.

PGLYRP-1 is known to be highly bactericidal [49], [50] and like OLFM-4, also appears to play a role in suppressing inflammation [51]. Thus, PGLYRP-1 could play a dual role in maintaining normal microflora and targeted killing of pathogenic bacteria. Mucin 5B has been shown to be required for respiratory tract health, and *muc5b^−/−^* mice exhibit severe morbidity and mortality due to an inability to clear routine debris (e.g., hair), leading to persistent bacterial infection of the lungs [44]. Additionally, human mucin 5B has demonstrated anti-HIV properties *in vitro*, presumably because of interactions between mucin 5B and the glycoproteins found in the viral envelope [52]. AGR2, one of the proteins that we found to be down-regulated in vaginal tissues, has been implicated in the production of mucus [53], [54], so the reduction in AGR2 may have affected levels of mucin 5B protein. Thus mucin 5B may also play two roles in the vagina by maintaining the proper bacterial flora as observed in airway epithelium and providing defense against viral pathogens like HIV.

**Figure 7:**
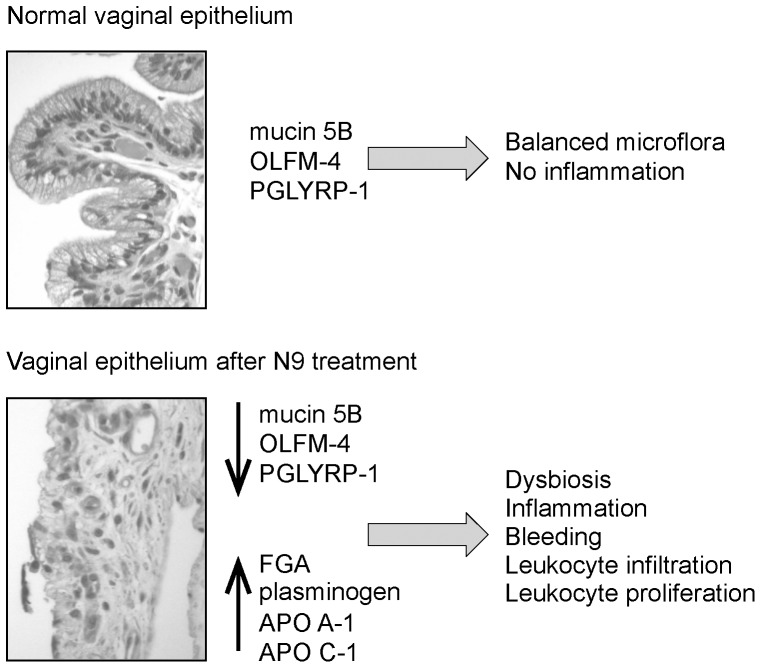
Model for the protective roles of mucin 5B, olfactomedin-4 (OLFM-4), and peptidoglycan recognition protein 1 (PGLYRP-1) in vaginal epithelium. In normal epithelium (top) these proteins maintain a balanced microflora and suppress inflammation. However, when exposed to toxic microbicides such as N9 (bottom) the epithelium becomes inflamed causing bleeding and release of serum proteins into the vaginal cavity. In addition, the microflora is altered leading to dysbiosis which could boost the inflammation response. The result is an influx and expansion of leukocytes that may be susceptible to HIV.

Given the diversity of the vaginal microflora, there must be a delicate balance between allowing for persistent growth of normal bacterial flora and suppression of an inflammatory response to remove them. N9 is known to alter the normal microflora of the vagina, including a marked decrease in several lactobacillus species with a subsequent increase in other potentially harmful bacterial species [55] that could induce an inflammatory response. We propose a model (**Figure 7**) in which these anti-inflammatory/protective proteins provide a stout epithelial barrier that support a balanced microbiota in the vagina; treatment with compounds such as N9, by inducing inflammation, causes damage to the vaginal epithelium, reduces the levels of these proteins leading to recruitment and dangerous exposure of HIV susceptible cells at the sites of tissue damage.

## Supporting Information

Figure S1 **Quantitative proteomic and RT-PCR analysis of mouse brush and tissue samples from mice treated with vehicle, buffergel, Tenofovir, and/or a range of N9 concentrations.** (A) ELISA analysis of day 7 mouse vaginal brush samples isolated from animals treated with either vehicle, Buffergel, or 1% Tenofovir. (B) mRNA expression analysis of CD166 antigen (CD166), peptidoglycan recognition protein 1 (PGLYRP-1), olfactomedin-4 (OLFM-4), mucin 5 subtype B (mucin 5B), and anterior gradient protein 2 homolog (AGR2) isolated from day 10 mouse vaginal tissues treated with vehicle, 1%, 4%, or 8% N9, Buffergel, or 1% Tenofovir. Statistical analysis was performed using a two-tailed Student's T-test of N9 samples compared to untreated controls at each time point (*** = p≤0.001, ** = p≤0.01, * = p≤0.05, NS = not significant).
(EPS)

Figure S2 **Analysis of mucin 5B expression in Vk2 cells. mRNA (left) and protein expression (right) of mucin 5B and olfactomedin-4 (OLFM-4) in untreated Vκ2 cells.** Shown are two replicate cDNA samples amplified using gene-specific RT-PCR oligonucleotides, and duplicate protein samples resolved by SDS-PAGE, then blotted to PDVF membranes and stained with anti-mucin 5B, anti-OLFM-4, or anti-actin antibodies. Glyceraldehyde 3-phosphate dehydrogenase (GAPDH) is included as a loading control for the RT-PCR.
(EPS)

Figure S3 **Viability of Vk2 cells treated with 0.001% N9 for 6, 24, and 48 hrs.** Viability testing was performed using CellTiter Glo which measures ATP levels in the cells.
(EPS)

Table S1 **List of RT-PCR primers.**
(DOCX)

Table S2 **List of biomarker candidates and their biological function.**
(DOCX)
