# Supplementary material for: New Candidate Biomarkers in the Female Genital Tract to Evaluate Microbicide Toxicity
Source: PLoS One. 2014 Oct 21;9(10):e110980. doi: 10.1371/journal.pone.0110980 (PMC4205019; doi:10.1371/journal.pone.0110980)
Supplement: Table S1 — List of RT-PCR primers. (DOCX) [file pone.0110980.s004.docx]

| **SI Table 1** | |
| --- | --- |
| **Gene to be amplified** | **5'-primer-3'** |
| Mouse PRAP1 forward (192 bp) | CTAGAGCCCTGGAGCCTCTT |
| Mouse PRAP1 reverse (192 bp) | TGGGATGGTCTATGCTGTCA |
| Mouse Ccl3 forward (163 bp) | GATGAATTGGCGTGGAATCT |
| Mouse Ccl3 reverse (163 bp) | CCTCTGTCACCTGCTCAACA |
| Mouse Ccl5 forward (157 bp) | TTGAACCCACTTCTTCTCTGG |
| Mouse Ccl5 reverse (157 bp) | CTGCTGCTTTGCCTACCTCT |
| Mouse Ccl7 forward (177 bp) | TCACGGTCCTAAGGGATAGG |
| Mouse Ccl7 reverse (177 bp) | GGGGAGAATTCTGCAGCTAA |
| Mouse OLFM4 forward (161 bp) | GCTGGAAGTGAAGGAGATGC |
| Mouse OLFM4 reverse (161 bp) | GGCGAATGCTAAGGACATTG |
| Mouse Muc5B forward (166 bp) | GATTCATGGGCATCTTCCTG |
| Mouse Muc5B reverse (166 bp) | CTGCGTGTGGTGAAGTCATT |
| Mouse Pglyrp1 forward (178 bp) | CATCAAGGGTGACCACACAG |
| Mouse Pglyrp1 reverse (178 bp) | CCGGTGTCCTTTGACTTCAT |
| Mouse cd166 forward (201 bp) | CTGACCCCAGTGGACTGTCT |
| Mouse cd166 (201 bp) | GTTGGTTTTCTGCTGTGCAA |
| Mouse ARG2 forward (155 bp) | GAGGGCAGACATCACTGGAC |
| Mouse AGR2 reverse (155 bp) | CTGCCTTCTGGTCTCCTGAC |
| Mouse GAPDH (229 bp) | CGCATCTTCTTGTGCAGTGCC |
| Mouse GAPDH (229 bp) | GGCCTTGACTGTGCCGTTGAATTT |
| Human GADPH primer forward (259 bp) | ACAGTCAGCCGCATCTTCTT |
| Human GADPH primer reverse (259 bp) | GACAAGCTTCCCGTTCTCAG |
| Human COX2 primer forward (150 bp) | TGCGGGAACACAACAGAGTA |
| Human COX2 primer reverse (150 bp) | GATAGCCACTCAAGTGTTGCAC |
| Human PGLYRP1 primer forward (170 bp) | TGTGCAGCACTACCACATGA |
| Human PGLYRP1 primer reverse (170 bp) | TGTAGTTGCCCATGAAGCTG |
| Human IL-8 primer forward (228 bp) | TAGCAAAATTGAGGCCAAGG |
| Human IL-8 primer reverse (228 bp) | AAACCAAGGCACAGTGGAAC |
| Human OLFM4 forward (184 bp) | CAGAGTGGAACGCTTGGAAT |
| Human OLFM4 reverse (184 bp) | CAGCTCGAAGTCCAGTTCAGT |
| Human Muc5b primer forward (89 bp) | GTGAGGAGGACTCCTGTCAAGT |
| Human Muc5b primer reverse (89 bp) | CCTCGCAGAAGGTGATGTTG |
