# Supplementary material for: New Candidate Biomarkers in the Female Genital Tract to Evaluate Microbicide Toxicity
Source: PLoS One. 2014 Oct 21;9(10):e110980. doi: 10.1371/journal.pone.0110980 (PMC4205019; doi:10.1371/journal.pone.0110980)
Supplement: Table S2 — List of biomarker candidates and their biological function. (DOCX) [file pone.0110980.s005.docx]

| **SI Table 2.** | |
| --- | --- |
| **Protein** | **Function*** |
| Apolipoprotein A-1 | Transport cholesterol from tissues to liver |
| Apolipoprotein C-1 | Binds free fatty acids |
| Fibrinogen, alpha polypeptide isoform 2 | Yield monomers that polymerize into fibrin and act as a cofactor in platelet aggregation |
| Plasminogen | Dissolve the fibrin of blood clots |
| Heat shock cognate 71 kDa protein | Act as a repressor of transcriptional activation |
| Corticosteroid-binding globulin | Major transport protein for glucocorticoids and progestins in the blood |
| Peptidoglycan recognition protein 1 | Anti-inflammatory, maintains normal microflora ,and kills pathogenic bacteria |
| Mucin 5, subtype B, tracheobronchial | Known to play a role in microbial defense in lung epithelium and has demonstrated antiviral activity *in vitro* |
| Destrin | Severs actin filaments (F-actin) and binds to actin monomers (G-actin) |
| Carbonyl reductase 3 | Has low NADPH-dependent oxidoreductase activity towards 4-benzoylpyridine and menadione |
| CD166 antigen | Cell adhesion molecule that binds to CD6 |
| Olfactomedin-4 | Known anti-inflammatory modulator in mouse gut epithelium |
| Anterior gradient protein 2 homolog | Required for MUC2 and MUC5B post-transcriptional synthesis and secretion. May play a role in the production of mucus |
| Calcium-activated chloride channel regulator 1 | involve in the regulation of mucus production and/or secretion by goblet cells |
| Protein-glutamine gamma-glutamyltransferase 2 | Catalyzes the cross-linking of proteins and the conjugation of polyamines to proteins |
| * The biological function of the proteins listed can be found on [www.uniprot.org](http://www.uniprot.org). | |
